# Constructing lncRNA-miRNA-mRNA networks specific to individual cancer patients and finding prognostic biomarkers

**DOI:** 10.1186/s12863-024-01251-9

**Published:** 2024-07-08

**Authors:** Shulei Ren, Wook Lee, Byungkyu Park, Kyungsook Han

**Affiliations:** Department of Computer Engineering, Inha University, 22212 Incheon, South Korea

**Keywords:** Competitive endogenous RNAs, lncRNA-miRNA-mRNA network, Prognostic biomarker, Cancer

## Abstract

**Background:**

The competitive endogenous RNA (ceRNA) hypothesis suggests that microRNAs (miRNAs) mediate a regulatory relation between long noncoding RNAs (lncRNAs) and messenger RNAs (mRNAs) which share similar miRNA response elements (MREs) to bind to the same miRNA. Since the ceRNA hypothesis was proposed, several studies have been conducted to construct a network of lncRNAs, miRNAs and mRNAs in cancer. However, most cancer-related ceRNA networks are intended for representing a general relation of RNAs in cancer rather than for a patient-specific relation. Due to the heterogeneous nature of cancer, lncRNA-miRNA-mRNA interactions can vary in different patients.

**Results:**

We have developed a new method for constructing a ceRNA network of lncRNAs, miRNAs and mRNAs, which is specific to an individual cancer patient and for finding prognostic biomarkers consisting of lncRNA-miRNA-mRNA triplets. We tested our method on extensive data sets of three types of cancer (breast cancer, liver cancer, and lung cancer) and obtained potential prognostic lncRNA-miRNA-mRNA triplets for each type of cancer.

**Conclusions:**

Analysis of expression patterns of the RNAs involved in the triplets and survival rates of cancer patients revealed several interesting findings. First, even for the same cancer type, prognostic lncRNA-miRNA-mRNA triplets can be different depending on whether lncRNA and mRNA show opposite or similar expression patterns. Second, prognostic lncRNA-miRNA-mRNA triplets are often more predictive of survival rates than RNA pairs or individual RNAs. Our approach will be useful for constructing patient-specific lncRNA-miRNA-mRNA networks and for finding prognostic biomarkers from the networks.

**Supplementary Information:**

The online version contains supplementary material available at 10.1186/s12863-024-01251-9.

## Background

A large portion of the human genome is transcribed to various RNAs, and approximately 98% of the RNAs are not translated into proteins [1]. The RNAs that do not code for proteins are collectively referred as non-coding RNAs (ncRNAs). Among the ncRNAs, microRNAs (miRNAs) are perhaps the best known regulatory ncRNAs. As small ncRNAS of $\sim$22 nucleotides, miRNAs often bind to the 3^′^ untranslated region (3^′^ UTR) of target mRNAs, resulting in translational repression and degradation of mRNAs [2]. Long non-coding RNAs (lncRNAs) are another type of ncRNAs with at least 200 nucleotides, and regulate gene expression in a variety of biological processes, including tumor proliferation, invasion and metastasis [3–5].

For the past decade, a variety of ncRNAs have been discovered and accumulating evidence has demonstrated that many miRNAs and lncRNAs are key regulators in the initiation and development of cancer. Salmena et al. [6] proposed a competitive endogenous RNA (ceRNA) hypothesis. The hypothesis suggests that miRNAs mediate a regulatory relation between lncRNAs and mRNAs which share similar miRNA response elements (MREs) to bind to the same miRNA. Although the ceRNA hypothesis is controversial and requires more work to validate, many studies have supported the hypothesis and demonstrated that dysregulation in lncRNA or miRNA is associated with several human diseases, including cancer [1, 7–10].

Motivated by accumulating evidence of the ceRNA hypothesis and the increasing number of newly discovered ncRNAs and mRNAs, several studies have been conducted recently to construct ceRNA networks of lncRNAs, miRNAs and/or mRNAs in cancer. Zhu et al. [11], for example, constructed a network of lncRNA-miRNA-mRNA triplets from miRNA-lncRNA associations and miRNA-mRNA associations. Jiang et al. [12] constructed a ceRNA network after calculating correlation coefficients of miRNA-mRNA and miRNA-lncRNA pairs. However, most cancer-related ceRNA networks are intended for representing a general relation of RNAs present in multiple cancer samples rather than for a patient-specific relation of RNAs. The biological functions of the regulatory ncRNAs are very diverse depending on the target molecules regulated by ncRNAs. In particular, cancer is a very heterogeneous disease, so lncRNA-miRNA-mRNA interactions can vary in different cancer patients.

In our recent study, we have developed a new method for constructing a patient-specific ceRNA network of lncRNAs, miRNAs and mRNAs and for finding prognostic biomarkers consisting of lncRNA-miRNA-mRNA triplets. With extensive samples of breast cancer, lung cancer and liver cancer, we constructed patient-specific ceRNA networks and found potential prognostic biomarkers consisting of lncRNA-miRNA-mRNA triplets. The rest of this paper presents our method for constructing patient-specific ceRNA networks and the results of the method in three types of cancer.

## Results

### ceRNA network

After the Z-test described in the previous section, a total of 5,133 lncRNA-miRNA-mRNA triplets were left for breast cancer, which involve 250 lncRNAs, 71 miRNAs, and 1,031 mRNAs. In a similar way, we obtained 345 and 1,804 lncRNA-miRNA-mRNA triplets for liver cancer and lung cancer, respectively.

**Fig. 1:**
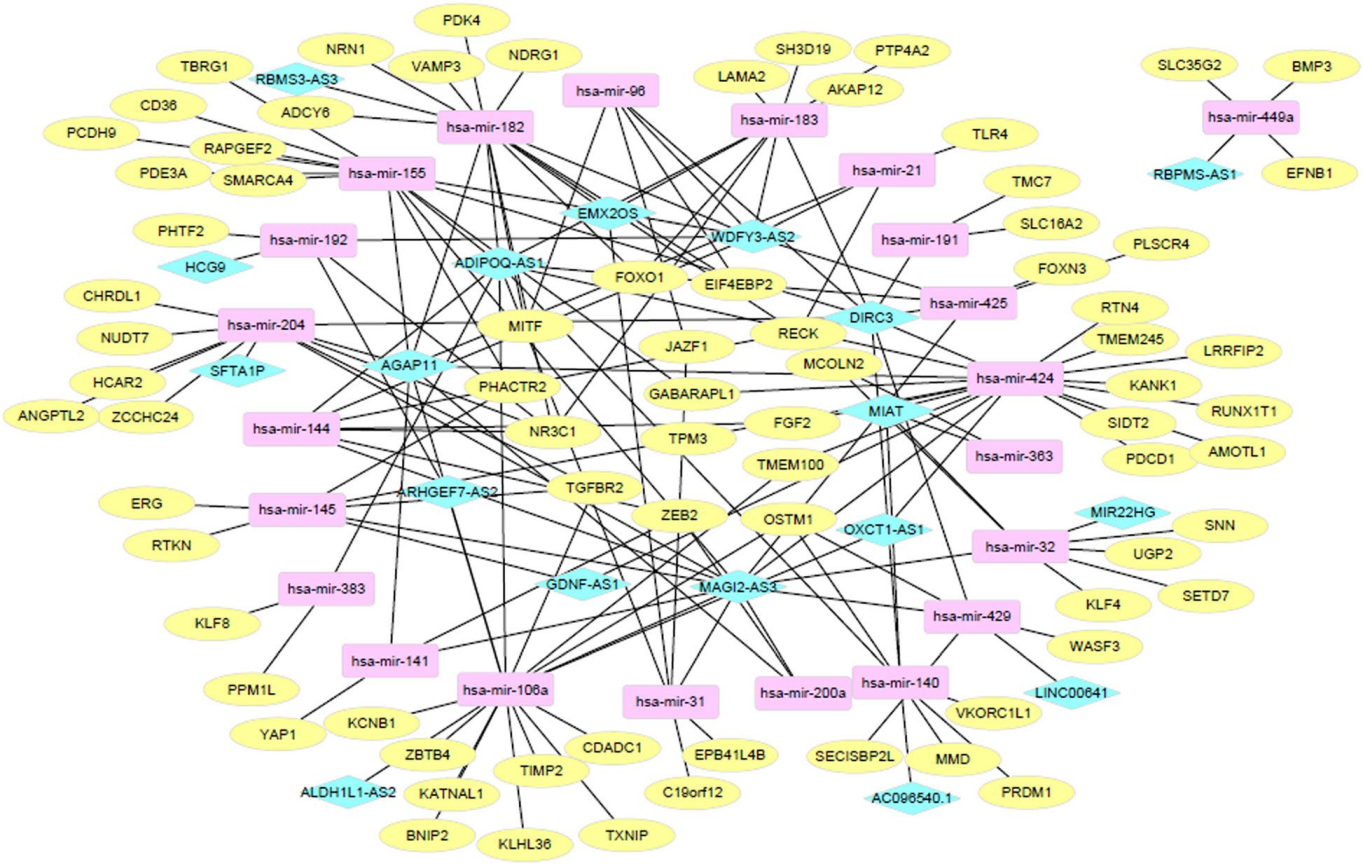
A ceRNA network of 183 lncRNA-miRNA-mRNA triplets in breast cancer. lncRNAs, miRNAs, and mRNAs are represented by diamonds, round rectangles, and ellipses, respectively

For each of the lncRNA-miRNA-mRNA triplets, we conducted the survival analysis of patients using the univariate Cox proportional hazards model. After selecting the triplets with *p*-value $< 0.01$ in the Cox proportional hazards model, 183 triplets were obtained as potential prognostic biomarkers for breast cancer. Eighteen lncRNAs, 22 miRNAs, and 76 mRNAs are involved in the 183 triplets. Figure 1 shows a ceRNA network constructed with the 183 triplets, which was visualized by Cytoscape v3.7.1.

The most connected node in the ceRNA network is hsa-mir-424, which is a miRNA participating in 24 triplets. Many studies have shown that hsa-mir-424 plays an important role in the development of tumor cells. Wang et al. [13] reported that HCC1806 and MDA-MB-468 cells transfected with miRNA mimics and found that the number of invasive cells in HCC1806 and MDA-MB-468 cells decreased significantly with the increase of time, and through further studies found that miR-424-5p can target DCLK1 to inhibit tumor cell proliferation, migration and invasion. The most frequently observed mRNA in the triplets is NR3C1, participating in 16 triplets. ER- subtype of breast cancer is known to be more aggressive than ER+ breast cancer and recurs easily. Glucocorticoid receptor (GR) encoded by GR gene (NR3C1) signaling initiates anti-apoptotic pathways in ER- breast cancer cells [14]. Among the lncRNAs, MAGI2-AS3 is the most frequently observed in the triplets. Many studies have shown that MAGI2-AS3 is a tumor suppressor gene in breast cancer. MAGI2-AS3 is known to inhibit breast cancer cell growth by targeting Fas and FasL signaling [15]. MAGI2-AS3 inhibits breast cancer metastasis by competing with miR-374a for PTEN [16].

For the ceRNA network of liver cancer, we obtained 91 triplets, which involve 14 lncRNAs, 32 miRNAs, and 53 mRNAs. The most frequent mRNA in the triplets was STK17B. Overexpression of STK17B is known to promote the proliferation and metastasis of liver cancer cells [17]. In the ceRNA network of liver cancer, STK17B interacts with the LINC00426 lncRNA via 5 different miRNAs. Among the lncRNAs in the ceRNA network, LINC00426 has the highest degree. LINC00426 is known to be related to immune infiltration level in liver cancer [18].

The ceRNA network of lung cancer includes 76 lncRNAs, 49 miRNAs, and 126 mRNAs. As one of key mRNAs in the ceRNA network of lung cancer, CCNE1 encodes cyclin E1 belonging to the highly conserved cyclin family. In the ceRNA network of lung cancer, CCNE1 interacts with SNHG1 and PVT1 via mir-497, which is known to down-regulate cyclin E1 to inhibit the growth of lung cancer cells [19]. In addition to that, both triplets (SNHG1_mir-497_CCNE1 and PVT1_mir-497_CCNE1) showed a significant impact on survival rates of lung cancer patients (Additional file 2). Li et al. [20] showed that SNHG1 and mir-497 have the reciprocal inhibitory relationship in lung cancer through experiments. Qin et al. [21] experimentally verified that PVT1 could act as the sponge of mir-497 in lung cancer cells.

### Analysis of differential gene expressions

We compared normal samples and breast cancer samples of TCGA using the R package edgeR [22], and selected lncRNAs, miRNAs, and mRNAs with |log2 fold change$| > 2$. After selecting RNAs with the adjusted *p*-value $<0.05$ in the log2 fold change, we obtained 1,029 lncRNAs, 86 miRNAs, and 2,163 mRNAs.

Two hundred forty-seven lncRNAs, 50 miRNAs, and 967 mRNAs involved in the 5,133 triplets from the Z-test were not differentially expressed between normal samples and breast cancer cells. For the non-differential lncRNAs and mRNAs, by searching the MalaCards database (https://www.malacards.org), we found that these lncRNAs and mRNAs are also related to breast cancer. For example, the |log2 fold change| of lncRNA LINC00472, H19 and PVT1 are all less than 2, but there are still studies showing that they are related to breast cancer. LINC00472 as a tumor suppressor gene, the high expression of LINC00472 in less aggressive breast tumors is more conducive to the prognosis of patients and also has a good response to adjuvant chemo- or hormonal therapy [23]. H19 has oncogenic potential in breast epithelial cells. In epithelial cells, GIT2 is essential for maintaining the epithelial status, and CYTH3 is essential for inducing mesenchymal phenotype, H19 can regulate GIT2 and CYTH3 expression through specific sponges of miR-200b/c and let-7b [24]. The transcription factor SOX2 enhances the transcription of PVT1 by combining with the promoter of PVT1, and the upregulated PVT1 promotes the proliferation and invasion of breast cancer cells via EMT [25].

### Prognostic biomarkers

Using the Cox proportional hazards model with lncRNA-miRMA-mRNA triplets selected by the Z-test, we selected 183, 91, and 278 potential prognostic lncRNA-miRNA-mRNA triplets for breast cancer, liver cancer, and lung cancer, respectively. For each of the triplets, we clustered cancer patients with respect to the expression levels (high vs. low) of the RNAs and performed the survival analysis of the cancer patients. Since there are 3 RNAs in each lncRNA-miRNA-mRNA triplet and their expression level is classified into either high or low, there are 8 combinations of the expression patterns of each triplet.

Although miRNAs play a key role in identifying lncRNA-miRNA-mRNA associations in cancer, the survival analysis showed that the expression level of miRNAs in the lncRNA-miRNA-mRNA triplet did not affect the survival rate of cancer patients. Interestingly, the survival rate of cancer patients were rather determined by the expression patterns of lncRNAs and mRNAs. Hence, we clustered cancer patients into two groups depending on the expression patterns of lncRNAs and mRNAs (Table 1).

- **Group 1:** patients with opposite expression patterns of lncRNA and mRNA (that is, one is high and the other is low)
- **Group 2:** patients with similar expression patterns of lncRNA and mRNA (that is, both are high or low)

In the above groups, the criteria for “high” and “low” gene expressions are the median of expression levels of the gene in all cancer samples of the same type. The reason for using the median instead of the average is that outliers in samples can result in severely unbalanced partitions of samples.

**Table 1:** Two groups of cancer patients with respect to expression patterns of lncRNA and mRNA

	lncRNA	mRNA
Group 1	High expression	Low expression
(Opposite expression patterns)	Low expression	High expression
Group 2	High expression	High expression
(Similar expression patterns)	Low expression	Low expression

**Fig. 2:**
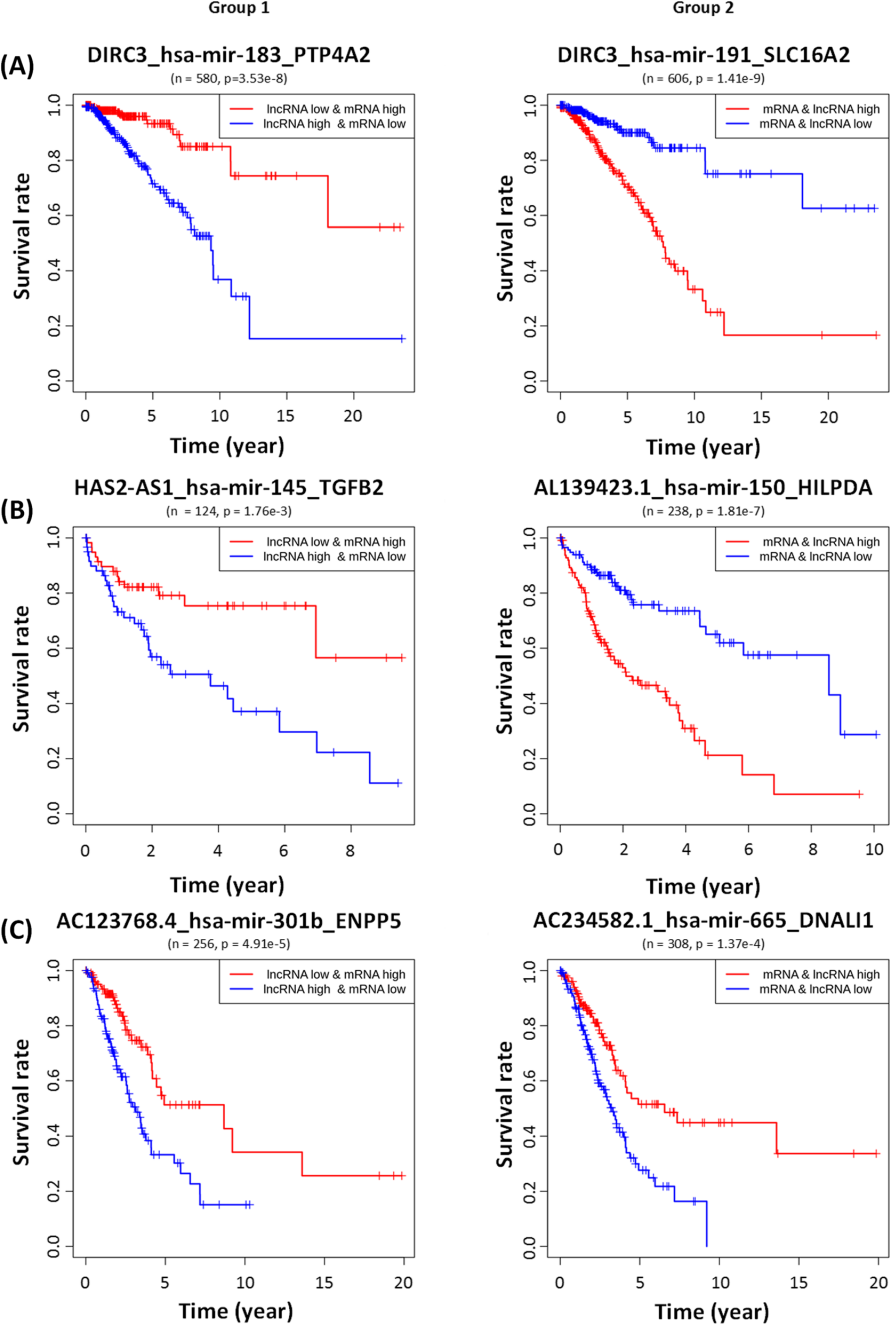
The survival rate of cancer patients with respect to prognostic lncRNA-miRNA-mRNA triplets found in our study. Group 1: patients with opposite expression patterns of lncRNAs and mRNAs in the lncRNA-miRNA-mRNA triplet. Group 2: patients with similar expression patterns of lncRNAs and mRNAs in the lncRNA-miRNA-mRNA. **A** The survival rate of BRCA patients with respect to two lncRNA-miRNA-mRNA triplets (DIRC3_hsa-mir-183_PTP4A2 and DIRC3_hsa-mir-191_SLC16A2). **B** The survival rate of LIHC patients with respect to two lncRNA-miRNA-mRNA triplets (HAS2-AS1_hsa-mir-145_TGFB2 and AL139423.1_hsa-mir-150_HILPDA). **C** The survival rate of LUAD patients with respect to two lncRNA-miRNA-mRNA triplets (AC123768.4_hsa-mir-301b_ENPP5 and AC234582.1_hsa-mir-665_DNALI1)

**Fig. 3:**
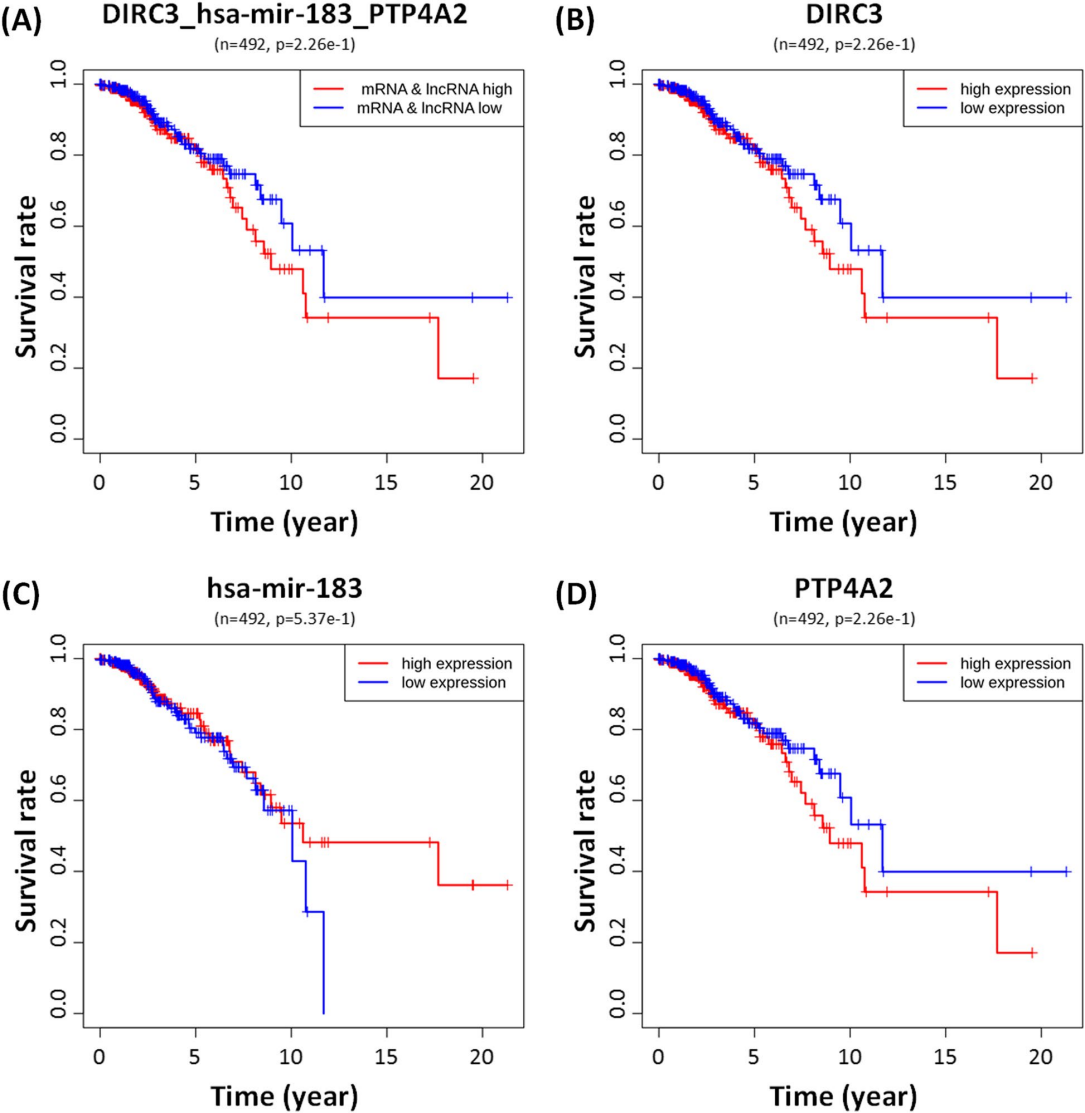
The survival rate of BRCA patients of Group 2. **A** The survival rate of BRCA patients with respect to DIRC3_hsa-mir-183_PTP4A2 triplet. **B** The survival rate of BRCA patients with respect to lncRNA DIRC3 alone. **C** The survival rate of BRCA patients of Group 2 with respect to miRNA hsa-mir-183 alone. **D** The survival rate of BRCA patients with respect to mRNA PTP4A2 alone. None of lncRNA, miRNA, and mRNA alone does not have predictive power of the survival rate of BRCA patients

Figure 2 compares the survival rate of two groups of patients with respect to lncRNA-miRNA-mRNA triplets in breast cancer, liver cancer and lung cancer. For example, breast cancer patients with low expression of DIRC3 and high expression of PTP4A2 show much a higher survival rate than those with high expression of DIRC3 and low expression of PTP4A2 (Fig. 2A) in Group 1. But, the DIRC3_hsa-mir-183_PTP4A2 triplet was not predictive of the survival rate of the patients of Group 2, which is a group of patients with similar expression patterns of lncRNA and mRNA (Fig. 3A). Individual RNAs involved in the DIRC3_hsa-mir-183_PTP4A2 triplet were not predictive of the survival rate, either, as shown in Fig. 3B, C and D. This result corroborates the previous study by Zhao et al. [26] that high expression of PTP4A2 is favorable in prognosis and that low expression of PTP4A2 is inversely correlated with the expression of genes involved in proliferation.

Additional file 3A shows the impact of different expression patterns of the HCG9_hsa-mir-192_PHTF2 triplet on the survival rate of breast cancer patients of two groups. The HCG9_hsa-mir-192_PHTF2 triplet showed the second smallest *p*-value (1.78e-6) in the survival analysis of breast cancer patients of Group 1. It is interesting to note that the HCG9_hsa-mir-192_PHTF2 triplet is very powerful in predicting the survival rate of Group 1, but loses its predictive power in Group 2. Our study showed that low expression of HCG9 and high expression of PHTF2 resulted in the high survival rate of breast cancer patients.

A similar observation was made for other triplets. For example, DIRC3_hsa-mir-191_TMC7 (*p*-value $=$ 1.13e-8 in Group 2) showed the second smallest *p*-values in the survival analysis of breast cancer patients (Additional file 3B). The DIRC3_hsa-mir-191_TMC7 triplet showed the predictive power of survival rate in Group 2 only. Elyakim et al. [27] found that hsa-mir-191 can trigger proliferation inhibition and apoptosis via up-regulating the expression of the target genes SOX4, IL1A and TMC7 in hepatocellular carcinoma. The results of our study correspond to the result of the study by Elyakim et al. [27].

For comparative purposes, we also ran the Cox proportional hazards model with the prognostic lncRNA-mRNA pairs obtained from our previous study [28], and each RNA involved in the triplets. Table 2 shows the result of the comparison with respect to the *p*-values from the Cox proportional hazards model. Table 3 summarizes the total number of lncRNA-miRNA-mRNA triplets in each group of cancer patients, which have a *p*-value < 0.05 from the Cox proportional hazards model. Details of the lncRNA-miRNA-mRNA triplets are given in Additional file 2.

**Table 2:** Comparison of *p*-values of lncRNA-miRNA-mRNA triplets, prognostic lncRNA-mRNA pairs obtained from our previous study [28], and each RNA involved in the triplets from the Cox proportional hazards model

Cancer	Group	lncRNA-miRNA-mRNA triplet	*p*-value from the Cox model				
			triplet	lncRNA-mRNA pair	lncRNA	mRNA	miRNA
BRCA	1	DIRC3_hsa-mir-183_PTP4A2	3.53e-8	2.21e-1	2.26e-1	5.37e-1	2.26e-1
		HCG9_hsa-mir-192_PHTF2	1.79e-6	6.14e-1	4.95e-1	8.77e-1	4.95e-1
		GDNF-AS1_hsa-mir-424_LRRFIP2	5.54e-6	3.45e-1	2.85e-1	3.92e-1	2.85e-1
	2	DIRC3_hsa-mir-191_SLC16A2	1.41e-9	3.35e-4	7.14e-1	2.86e-1	7.14e-1
		DIRC3_hsa-mir-191_TMC7	1.13e-8	3.06e-3	5.30e-1	3.96e-2	5.30e-1
		DIRC3_hsa-mir-424_RTN4	4.40e-8	2.85e-2	2.03e-1	4.30e-1	2.03e-1
LIHC	1	HAS2-AS1_hsa-mir-145_TGFB2	1.76e-3	7.78e-1	1.37e-1	1.27e-1	1.37e-1
		AL139423.1_hsa-mir-424_TSC22D2	4.28e-3	4.10e-4	5.25e-4	9.49e-2	5.25e-4
		AL139423.1_hsa-mir-454_SOX4	7.60e-3	9.14e-1	3.13e-4	8.16e-1	3.13e-4
	2	AL139423.1_hsa-mir-150_HILPDA	1.81e-7	4.36e-1	5.72e-1	2.91e-1	5.72e-1
		AL139423.1_hsa-mir-122_SLC2A3	1.32e-5	3.76e-3	6.84e-2	7.66e-1	6.84e-2
		AL139423.1_hsa-mir-195_PAFAH1B2	1.24e-4	3.57e-1	1.00e-2	3.01e-1	1.00e-2
LUAD	1	AC123768.4_hsa-mir-301b_ENPP5	4.91e-5	6.41e-1	3.50e-1	7.09e-1	3.50e-1
		MZF1-AS1_hsa-mir-665_DNALI1	1.27e-3	3.53e-1	1.81e-1	8.39e-1	1.81e-1
		AGAP11_hsa-mir-424_CPEB3	1.48e-3	6.02e-2	1.23e-1	9.62e-1	1.23e-1
	2	SOX2-OT_hsa-mir-301b_ENPP5	7.17e-5	6.21e-1	7.73e-1	7.09e-1	5.28e-1
		AC234582.1_hsa-mir-665_DNALI1	1.37e-4	8.78e-1	8.00e-1	9.42e-1	9.17e-1
		AC004832.1_hsa-mir-17_ENPP5	4.47e-3	4.05e-3	1.33e-1	8.48e-1	1.33e-1

**Table 3:** The total number of lncRNA-miRNA-mRNA triplets in each group of cancer patients, which have a *p*-value < 0.05 from the Cox proportional hazards model. Group 1: patients with opposite expression patterns of lncRNA and mRNA (one is high and the other is low) Group 2: patients with similar expression patterns of lncRNA and mRNA (both are either high or low). See the prognostic biomarkers section for the definition of high and low expressions

Cancer	#potential prognostic triplets	
	Group 1	Group 2
Breast cancer	30	135
Liver cancer	29	15
Lung cancer	66	135

## Discussion

Although the ceRNA hypothesis needs more work to unveil the details of the relation among lncRNAs, miRNAs and mRNAs, many studies have demonstrated that lncRNAs and mRNAs with similar miRNA response elements (MREs) compete for binding to the same miRNA and that dysregulation in lncRNA or miRNA is related with several human diseases, including cancer. So far, several studies have been conducted to construct a network to show the relation among lncRNAs, miRNAs and/or mRNAs. However, most cancer-related ceRNA networks represent a general relation of RNAs in multiple cancer samples rather than a patient-specific relation of RNAs. Due to the heterogeneous nature of cancer, lncRNA-miRNA-mRNA interactions can vary in different patients.

The potential prognostic biomarkers derived from the lncRNA-miRNA-mRNA networks constructed in our work revealed a few interesting findings. First, even for the same cancer type, prognostic lncRNA-miRNA-mRNA triplets can be different depending on whether lncRNA and mRNA show opposite or similar expression patterns. Second, prognostic lncRNA-miRNA-mRNA triplets are often more predictive of survival rates than RNA pairs or individual RNAs.

## Conclusion

We have developed a new method for constructing a ceRNA network of lncRNAs, miRNAs and mRNAs, which is specific to an individual cancer patient and for finding prognostic biomarkers consisting of lncRNA-miRNA-mRNA triplets. We tested the method on extensive data sets of breast cancer, liver cancer, and lung cancer and obtained potential prognostic lncRNA-miRNA-mRNA triplets for each type of cancer. Evaluation of the results of testing showed that our approach is useful for generating patient-specific ceRNA networks and that potential prognostic lncRNA-miRNA-mRNA triplets are often more predictive of survival rates than RNA pairs or individual RNAs. Although preliminary, our approach will help us better understand lncRNA-miRNA-mRNA interactions in cancer patients and find effective and safe treatment for individual patients.

## Methods

In this section, we describe our method for constructing patient-specific ceRNA networks of lncRNA-miRNA-mRNA triplets and for deriving potential prognostic triplets.

### Data collection

For primary tumor samples of three types, breast invasive carcinoma (BRCA), lung adenocarcinoma (LUAD), and liver hepatocellular carcinoma (LIHC), we collected RNA-seq gene expression data of lncRNAs, mRNAs and miRNAs from the Cancer Genome Atlas (TCGA) data portal [29]. Normal samples of each type of cancer were also extracted from the TCGA data portal. As gene names in TCGA are represented by Ensembl ID, we obtained annotation files obtained from Ensembl (http://www.ensembl.org).

Since there has been no publicly available resource providing a large amount of data on lncRNA-miRNA-mRNA associations, we derived initial lncRNA-miRNA-mRNA triplets by merging lncRNA-miRNA associations and mRNA-miRNA associations. We collected both experimentally validated or computationally predicted lncRNA-miRNA associations from the miRcode database (http://www.mircode.org) [30], and selected the lncRNA-miRNA associations that are shared by starBase (http://starbase.sysu.edu.cn) [31]. mRNAs targeted by miRNAs were obtained by selecting mRNAs which are common to the miRDB (http://mirdb.org) [32], miRTarBase (http://mirtarbase.mbc.nctu.edu.tw) [33], and TargetScan (http://www.targetscan.org/vert_72) [34] databases. Additional file 1 shows 29,032 lncRNA-miRNA associations and 10,048 mRNA-miRNA associations, which were used in deriving the initial lncRNA-miRNA-mRNA triplets. Table 4 shows the number of tumor and normal samples, initial lncRNA-miRNA-mRNA triplets, and RNAs involved in the triplets.

**Table 4:** Number of samples, initial lncRNA-miRNA-mRNA triplets, and RNAs of each type which are involved in the triplets

Cancer type	#tumor samples	#normal samples	#triplets	#lncRNAs	#miRNAs	#mRNAs
BRCA	1,079	104	1,222,492	1,738	73	3,377
LIHC	369	50	959,254	1,334	71	3,326
LUAD	509	20	1,149,792	1,620	72	3,364

### Constructing ceRNA networks specific to individual patients

Several methods have been developed to characterize relations among lncRNAs, miRNAs, and mRNAs in cancer [1, 7–10]. To derive a ceRNA network in breast cancer, Paci et al. [35] computed the sensitivity of correlation between lncRNAs and mRNAs, which is defined by the difference between the correlation of lncRNAs and mRNAs and their partial correlation with respect to shared miRNAs. The ceRNA network constructed in their work is not intended for representing lncRNA-miRNA-mRNA relations specific to a single patient, but rather for general lncRNA-miRNA-mRNA relations in cancer. Zhang et al. [36] found cancer-related miRNA-lncRNA pairs by calculating the difference of the Pearson correlation coefficients (PCC) of lncRNAs and miRNAs between normal samples and tumor samples.

**Fig. 4:**
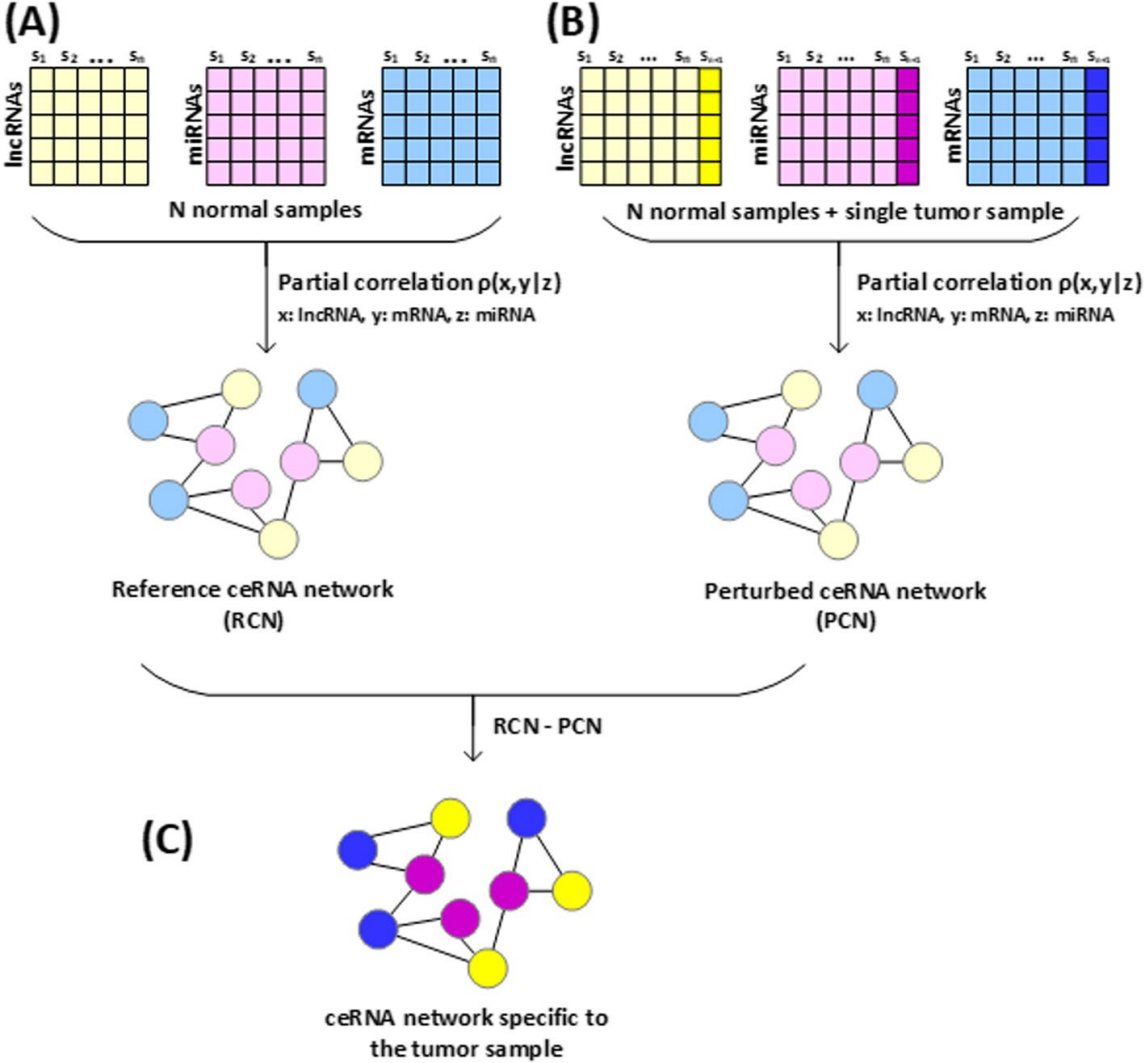
The procedure of constructing a ceRNA network specific to a tumor sample. **A** A reference ceRNA network is first constructed by computing partial correlations between lncRNAs and mRNAs mediated by miRNAs in N normal samples. **B** A perturbed ceRNA network is obtained by adding a tumor sample of the patient to the N normal samples. **C** A ceRNA network specific to the tumor sample is obtained by subtracting the reference network from the perturbed network

In our study, we considered mRNA-miRNA associations as well as miRNA-lncRNA associations to construct a patient-specific ceRNA network. Thus, the ceRNA network consist of lncRNA-miRNA-mRNA triplets. With a single sample alone, a network of lncRNA-miRNA-mRNA triplets specific to the sample cannot be constructed because a network requires multiple samples to compute the inter-relations among lncRNAs, miRNAs, and mRNAs. Therefore, we first constructed a reference ceRNA network with normal samples (Fig. 4A). Each edge in the reference ceRNA network represents a partial correlation coefficient ($\rho$), which was computed by Eq. (1) based on the expression levels of lncRNAs, mRNAs and miRNAs.

1 $$\begin{aligned} \rho (x, y|z){} & {} = \dfrac{PCC(x, y) - PCC(x, z)PCC(y, z)}{\sqrt{1 - PCC^{2}(x, z)}\sqrt{1 - PCC^{2}(y, z)}}\nonumber \\ \text {where} \,\,\,\,{} & {} \text {x: lncRNA}\nonumber \\{} & {} \text {y: mRNA} \nonumber \\{} & {} \text {z: miRNA} \end{aligned}$$

After constructing a reference ceRNA network, we added a single tumor sample to the normal samples, and recomputed partial correlation coefficients with $n+1$ samples to construct a perturbed ceRNA network (Fig. 4B). By subtracting the reference network ($\rho _{n}$) from the perturbed network ($\rho _{n+1}$) by Eq. (2), we obtained a set of differential partial correlation coefficients ($\Delta \rho$). A ceRNA network specific to the tumor sample is defined by the differential partial correlation coefficients (Fig. 4C).

2 $$\begin{aligned} \Delta\rho (x, y|z){} & {} = |\rho _{n +1}(x, y|z) - \rho _{n}(x, y|z)| \nonumber \\ \text {where} \,\,\,\,{} & {} \text {x: lncRNA} \nonumber \\{} & {} \text {y: mRNA} \nonumber \\{} & {} \text {z: miRNA} \nonumber \\{} & {} \text {n: number of samples} \end{aligned}$$

### Finding potential biomarkers of lncRNA-miRNA-mRNA triplets for prognosis

Cancer is typically associated with dysregulation of multiple genes rather than a single gene. Unlike biomarkers consisting of individual genes or combination of genes, a network biomarker involves not only multiple genes but also their interactions. Several studies, including our previous study [28], have shown that network biomarkers are more powerful than single genes in detecting the occurrence of cancer and in predicting prognosis of cancer.

**Fig. 5:**
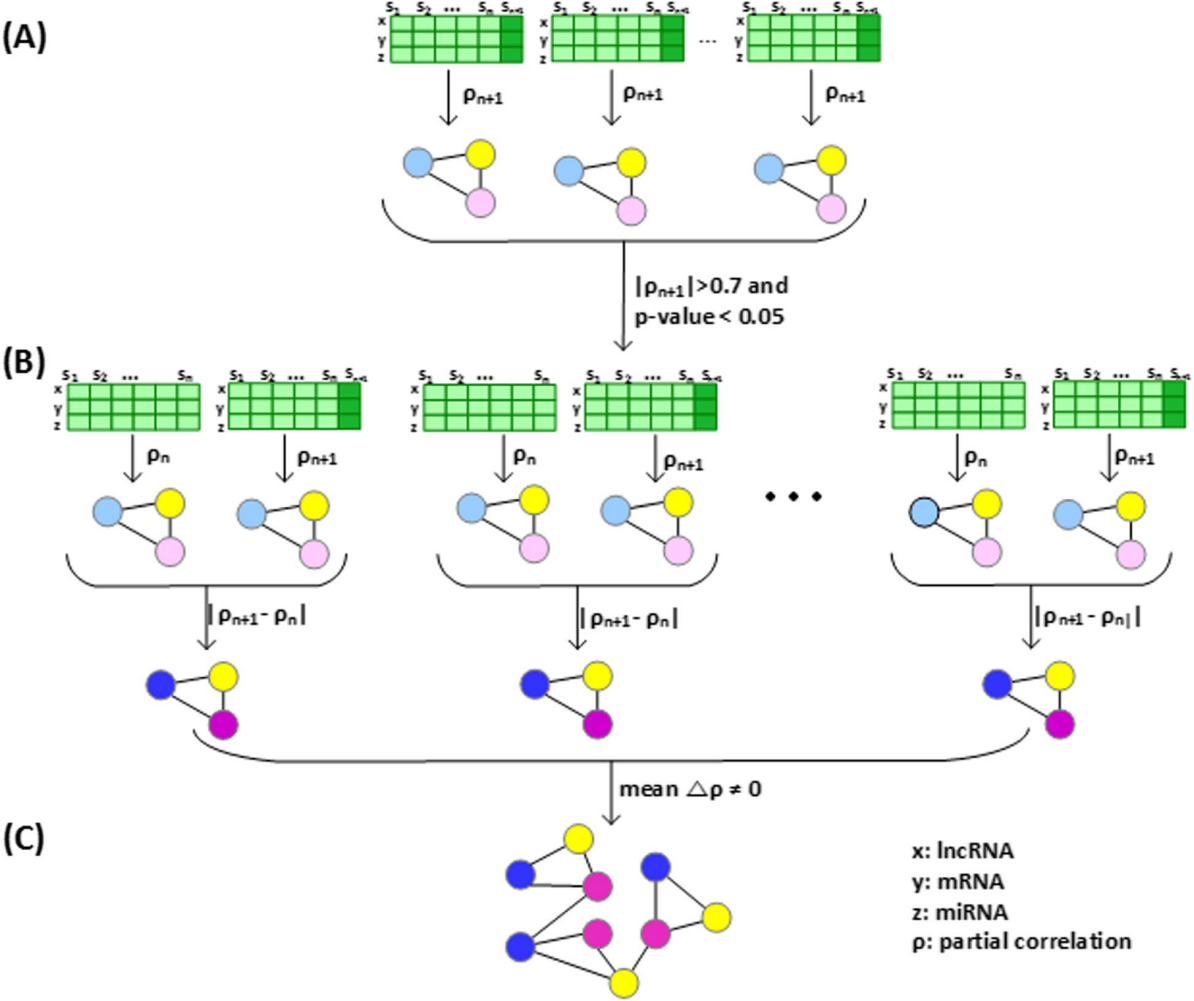
The process of performing the Z-test for lncRNA-miRNA-mRNA triplets. **A** Compute partial correlations $\rho$ between lncRNAs and mRNAs mediated by miRNAs after adding each tumor sample to normal samples, and select those partial correlations whose absolute values $>0.7$ and *p*-value $< 0.05$. **B** For each tumor sample, compute the differential partial correlation $\Delta \rho$ of the lncRNA-miRNA-mRNA triplets from the reference samples. **C** Select the lncRNA-miRNA-mRNA triplets with the mean $\Delta \rho \ne 0$

Since the primary focus of this work is to construct a patient-specific ceRNA network of lncRNA-miRNA-mRNA triplets, we attempted to find potential prognostic triplets from the network. To select patient-specific lncRNA-miRNA-mRNA associations, we performed a Z-test in the following way. The overall process is also shown in Fig. 5.

1. Compute partial correlations $\rho$ between lncRNAs and mRNAs mediated by miRNAs after adding each tumor sample to normal samples, and select those partial correlations whose absolute values $>0.7$ and *p*-value $< 0.05$ (Fig. 5A).
2. For each tumor sample, compute the differential partial correlation $\Delta \rho$ from the reference samples (Fig. 5B).
3. Select the lncRNA-miRNA-mRNA triplets with the mean $\Delta \rho \ne 0$ (Fig. 5C).

To find potential prognostic biomarkers, we performed the univariate Cox regression with respect to the expression levels of lncRNAs, miRNAs and mRNAs. The expression levels of the RNAs are classified into either high or low based on the median expression level of RNAs of each type in tumor samples. For the lncRNA-miRNA-mRNA triplets, there are a total of eight possible combinations of expression levels.

### Supplementary Information

Additional file 1: 29,032 lncRNA-miRNA associations and 10,048 mRNA-miRNA association from databases. Raw data for initial triplets in Table 4.

Additional file 2: Potential prognostic triplets for breast cancer, liver cancer, and lung cancer. The p-value of the triplet from the Cox model and the number of samples of each group.

Additional file 3: The second smallest p-value from the Cox model in breast cancer. (A) Kaplan-Meier plot of the HCG9_hsa-mir-192_PHTF2 triplet in Groups 1 and 2. The HCG9_hsa-mir-192_PHTF2 triplet shows the predictive power of survival rate in Group 1 only. (B) Kaplan-Meier plot of the DIRC3_hsa-mir-191_TMC7 triplet in Groups 1 and 2. The DIRC3_hsa-mir-191_TMC7 triplet shows the predictive power of survival rate in Group 2 only.

## Data Availability

Additional files are available at http://bclab.inha.ac.kr/ceRNA.

## Abbreviations

ceRNA: Competitive endogenous RNA
miRNA: MicroRNA
lncRNA: Long noncoding RNA
mRNA: Messenger RNA
MRE: miRNA response elements
BRCA: Breast invasive carcinoma
LUAD: Lung adenocarcinoma
LIHC: Liver hepatocellular carcinoma
PCC: Pearson correlation coefficient
GR: Glucocorticoid receptor
