# Supplementary figures and images for: Constructing lncRNA-miRNA-mRNA networks specific to individual cancer patients and finding prognostic biomarkers
Source: BMC Genom Data. 2024 Jul 8;25(Suppl 1):67. doi: 10.1186/s12863-024-01251-9 (PMC11232193; doi:10.1186/s12863-024-01251-9)

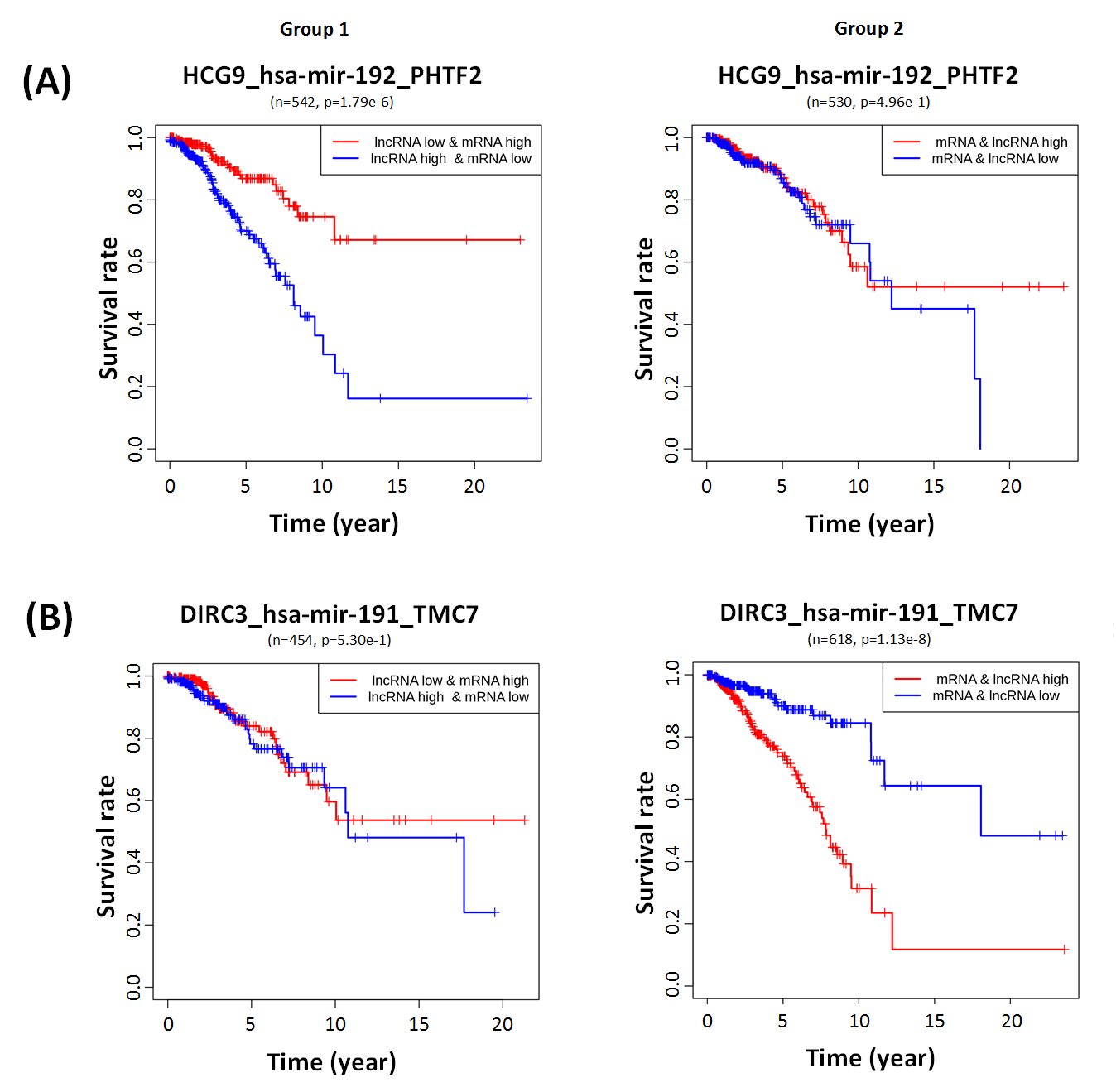

Supplement: Supplementary file 3 — Additional file 3: The second smallest p-value from the Cox model in breast cancer. (A) Kaplan-Meier plot of the HCG9_hsa-mir-192_PHTF2 triplet in Groups 1 and 2. The HCG9_hsa-mir-192_PHTF2 triplet shows the predictive power of survival rate in Group 1 only. (B) Kaplan-Meier plot of the DIRC3_hsa-mir-191_TMC7 triplet in Groups 1 and 2. The DIRC3_hsa-mir-191_TMC7 triplet shows the predictive power of survival rate in Group 2 only. [file 12863_2024_1251_MOESM3_ESM.png]
